# Collapsing focal segmental glomerulosclerosis following long-term treatment with oral ibandronate: case report and review of literature

**DOI:** 10.1186/s12885-015-1536-y

**Published:** 2015-07-22

**Authors:** Ning Jia, Fionnuala C. Cormack, Bin Xie, Zita Shiue, Behzad Najafian, Julie R. Gralow

**Affiliations:** 1 Department of Medical Oncology, Peking Union Medical College Hospital, Peking Union Medical College and Chinese Academy of Medical Sciences, Beijing, 100730 China; 2 Division of Nephrology, Harborview Medical Center, University of Washington, Seattle, WA 98195 USA; 3 Division of Oncology, Department of Medicine, Seattle Cancer Care Alliance, University of Washington, Seattle, WA 98109 USA; 4 Department of Medicine, University of Washington, Seattle, WA 98195 USA; 5 Department of Pathology, University of Washington, Seattle, WA 98195 USA

**Keywords:** Collapsing glomerulopathy, Focal segmental glomerulosclerosis, Bisphosphonates, Ibandronate

## Abstract

**Background:**

Renal toxicity has been reported with bisphosphonates such as pamidronate and zolidronate but not with ibandronate, in the treatment of breast cancer patients with bone metastasis. One of the patterns of bisphosphonate-induced nephrotoxicity is focal segmental glomerulosclerosis (FSGS) or its morphological variant, collapsing focal segmental glomerulosclerosis (CFSGS).

**Case presentation:**

We describe a breast cancer patient who developed heavy proteinuria (protein/creatinine ratio 9.1) and nephrotic syndrome following treatment with oral ibandronate for 29 months. CFSGS was proven by biopsy. There was no improvement 1 month after ibandronate was discontinued. Prednisone and tacrolimus were started and she experienced a decreased in proteinuria.

**Conclusion:**

In patient who develops ibandronate-associated CFSGS, proteinuria appears to be at least partially reversible with the treatment of prednisone and/or tacrolimus if the syndrome is recognized early and ibandronate is stopped.

## Background

Bisphosphonates are widely used in the treatment of patients with metastatic bone diseases. They play an important role in decreasing bone morbidity, preventing bone complications and managing bone pain. Furthermore, emerging evidence suggests that bisphosphonates may potentially inhibit angiogenesis and tumor cell invasion, induce apoptosis in tumor cells and have immunomodulatory effects [1, 2]. They have been increasingly studied in the adjuvant setting of breast cancer treatment and have showed some promising effects [3–5].

Bisphosphonates can be administered either orally or intravenously. Oral ibandronate was evaluated in the German Intergroup Node-Positive (GAIN) study, in which patients receiving one of two dose-dense chemotherapy regimens were randomized to 2 years of oral ibandronate versus placebo [6]. The first interim efficacy analysis was also presented at San Antonio Breast Cancer Symposium in December 2011. There was no difference in 3 year disease free survival or overall survival. The Southwest Oncology Group (SWOG) S0307 trial is a comparison of three different bisphosphonates in the adjuvant breast cancer setting. This trial randomized 6,000 women with stage I- III breast cancer receiving standard adjuvant therapy to oral clodronate (1600 mg, daily) versus oral ibandronate (50 mg, daily) versus zoledronate (4 mg intravenously, monthly for 6 months, then every 3 months), all for 3 years duration. This trial recently closed to accrual and will provide further data on the utility of bisphosphonates in the adjuvant setting.

Drug-related side effects are limiting factors to the use of bisphosphonates. Bisphosphonates have been associated with deterioration of renal function and histopathological changes in the kidney. Available data suggest that pamidronate and zolidronate, but not ibandronate, are associated with nephrotoxicity in the treatment of patients with malignant disease [7]. One of the patterns of nephrotoxicity with bisphophonates is the development of the focal segmental glomerulosclerosis (FSGS) or its morphological variant the collapsing focal segmental glomerulosclerosis (CFSGS), which typically presents with nephrotic syndrome and renal insufficiency [7]. To our knowledge, no patients with CFSGS and nephrotic syndrome associated with ibandronate have been described in the literature before.

## Case presentation

A 44-year-old Caucasian female with a family history of breast cancer and multiple myeloma was seen in clinic for right breast, stage I, triple negative invasive ductal carcinoma. She had undergone a lumpectomy followed by adjuvant dose-dense Adriamycin, Cyclophosphamide (AC) and paclitaxel chemotherapy and radiation. She was enrolled in the SWOG S0370 study and randomized to the oral ibandronate arm and had been taking oral ibandronate 50 mg once daily since December 2008 with stop date scheduled for December 2011. In May 2011, she presented with new onset lower extremity edema. Laboratory tests at the time revealed heavy proteinuria with a protein/creatinine ratio 9.1 (g/g), hypoalbuminaemia (2.1 g/l) and hypercholesterolaemia (14 mmol/l) with no hematuria and normal serum creatinine (0.7 mg/dl) which had been consistently normal before. She had no hypertension history. A diagnosis of nephrotic syndrome was made.

Serological evaluation for HIV, hepatitis C, hepatitis B virus, antibodies to nuclear antigens (ANA), rheumatoid factor, anti-neutrophil cytoplasmic antibody (anti-PR3 and anti-MPO), anti cardiolipin, and anti beta 2 glycoprotein 1 were negative. Values of serum complement (total, C3 and C4), free light chain (kappa and lambda), protein electrophoresis, immnofixation,beta 2 microglobulin and immunoglobulin (A, G and M) were within normal range. There were no spikes by urine and serum protein electrophoresis.

**Fig. 1:**
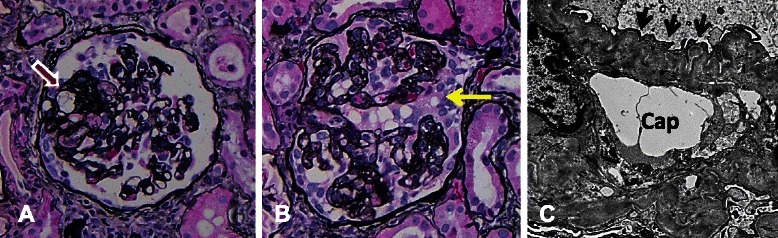
**a** A glomerulus with FSGS. The red arrow marks the segmentally sclerosed region with obliteration of capillary tuft, hyaline deposition and adhesion to Bowman’s capsule. Jones methenamine silver, 40x objective. **b** A glomerulus with collapsing variant of FSGS. There is almost diffuse collapse of capillary loops with proliferation of overlying epithelial cells (yellow arrow). Jones methenamine silver, 40x objective. **c** An electron micrograph shows extensive effacement of foot processes (black arrows) over a capillary loop (cap) with corrugated glomerular basement membrane. 4800 X magnification

A renal biopsy was performed and specimens were sent for light, immunofluorescent and electron microscopy. The biopsy consisted of portions of cortex and medulla, containing 10 to 15 glomeruli per level section. None of the glomeruli were globally sclerosed. About 13–40 % of glomeruli per level section showed segmental obliteration of capillary loops with accumulation of extracellular matrix, hyaline or foam cells in the sclerotic regions and adhesion to Bowman’s capsule, consistent with focal and segmental glomerulosclerosis (FSGS) (Fig. 1a). Up to two glomeruli with FSGS showed segmental collapse of capillary tufts with proliferation of overlying epithelial cells, characteristic of collapsing glomerulopathy (Fig. 1b). No additional lesion was found in the glomeruli. The rest of renal parenchyma showed mild interstitial fibrosis and tubular atrophy, mild and scattered predominantly mononuclear interstitial, inflammatory infiltrate, diffuse, mild interstitial edema and occasional tubulitis. Arterioles were unremarkable, but interlobular arteries showed moderate intimal fibrosis. Immunofluorescence studies were unremarkable with no significant glomerular staining for IgG, IgA, IgM, C3, C1q, fibrinogen, kappa or lambda light chains or albumin. Electron microscopy showed extensive effacement of foot processes involving about 60 to 70 % of capillary surface area (Fig. 1c). No electron dense deposit was identified. After the diagnosis of nephrotic syndrome, ibandronate was discontinued. She initially underwent treatment with lisinopril, simvastatin and furosemide. However, after one month, she had no improvement of protenuria. Prednisone was started at 60 mg daily. About one month later, urine protein/creatinine ratio was reduced to 4.9 g/g, thus indicative of some improvement but still within the nephrotic range. Therefore, a calcineurin inhibitor, tacrolimus 1 mg twice daily was started in August 2011 and continued through October 2013. Prednisone was tapered off after 7 months in March 2012. Creatinine was relatively stable throughout treatment and Estimated Glomerular Filtration Rate (eGFR) calculated by Modification of Diet in Renal Disease (MDRD) equation was normal. She is currently on lisinopril 40 mg daily with mild proteinuria (protein/creatinine ratio of 0.5) at the time of this report.

## Discussion

We report a rare case of collapsing FSGS and nephrotic syndrome associated with the administration of oral ibandronate. Ibandronate is a single nitrogen-containing bisphosphonate with higher comparative protein binding and shorter comparative renal tissue half life than other bisphosphonates. It is Food and Drug Administration approved for treatment of post-menopausal osteoporosis (PMO). Outside the United States, ibandronate is approved by the European Union for the treatment of PMO and malignancy-associated bone disease.

Ibandronate appears to have a safer renal profile with no evidence of nephrotoxicity. Intravenous ibandronate given over 15 min, 60 min or 15–30 s did not have renal toxicity, even in patients with preexisting hypertension or diabetes, although follow up is short [8–10]. Fourteen patients who received a median of 16 monthly infusions of ibandronate have no renal toxicity [11]. With 44 patients and 524 cycles of oral idandronate with a median of 12 monthly cycles, there is no deterioration of renal function [12]. In patients with preexisting renal impairment, there is no acute nephrotoxicity with ibandronate [13]. Results from clinical trials indicate that the renal safety profile of intravenous and oral ibandronate is similar to that of placebo for up to 4 years treatment in metastatic breast cancer [14, 15].

The renal biopsy findings in our patient displayed FSGS with collapsing features and extensive foot process effacement, consistent with significant podocyte injury. Pamidronate toxicity targets podocytes, while some cases of pamidronate-associated toxic acute tubular necrosis (ATN) have been reported [16]. Zoledronate appears to mainly target tubular epithelium, resulting in a pattern of toxic ATN, but zoledronate-associated collapsing FSGS has also been reported [17]. Therefore, renal injury of intravenous bisphosphonates is likely to be very complex and overlap between different agents. We did not identify significant acute tubular injury in the current biopsy.

The renal tolerability of oral bisphosphonates is relatively good. However, nephrotoxicity has been reported with oral bisphosphonates. Miura et al. reported a single case of massive proteinuria and acute renal failure after alendronate administration in a patient with focal segmental glomerulosclerosis [18]. Stratton et al. reported two cases of relapse of steroid-dependent nephrotic syndrome triggered by etidronate [19]. Proteinuria is thus a known feature of bisphosphonate-associated nephrotoxicity. In many cases, nephrotic syndrome associated with bisphosphonate is partially or completely reversible following discontinuation of bisphosphonate [18–20].

The frequency of proteinuria in breast cancer patients with metastatic bone disease receiving intravenous ibandronate was similar to those receiving placebo or no clinical relevant changes from baseline in 2 randomized trials [10, 21]. However the numbers of patients were limited and the study periods were only 3 and 6 months respectively. Transient proteinuria after the treatment of intravenous ibandronate for metastatic breast cancer has been reported before [22].

The American Society of Clinical Oncology 2011 Clinical Practice Guideline Update committee recommends that serum creatinine should be monitored before each dose of pamidronate or zoledronic acid per FDA-approved labeling [23]. Serum calcium, electrolytes, phosphate, magnesium, and hematocrit/hemoglobin should also be monitored regularly. Therefore, since proteinuria was not monitored commonly in the patients treated with long-term intravenous and oral ibandronate before, the renal toxicity of ibandronate could be underestimated.

The etiology for the difference in nephrotoxic potential is not entirely clear, but could relate to pharmacokinetic differences. Ibandronate is more highly protein bound, which may limit renal exposure of free drug. The renal tissue half-life of ibandronate is much shorter than zoledronate [24].

## Conclusion

We report an unusual case of oral ibandronate-associated CFSGS and nephrotic syndrome. Proteinuria appears to be partially reversible with the treatment of prednisone and/or tacrolimus if the syndrome is recognized early and ibandronate is stopped. We did not have a follow up biopsy to confirm if the observed improvement in proteinuria was associated with histological amelioration of FSGS or foot process effacement. We recommend monitoring proteinuria for earlier detection of worsening renal function in patients with long-term treatment of oral ibandronate. Renal biopsy is a key procedure for diagnosis and treatment.

## Consent

Written informed consent was obtained from the patient for publication of this case report and any accompanying images. A copy of the written consent is available for review by the editor of this journal.

## Abbreviations

FSGS: Focal segmental glomerulosclerosis
CFSGS: Collapsing focal segmental glomerulosclerosis
GAIN: The German Intergroup Node-Positive
SWOG: The Southwest Oncology Group
AC: Adriamycin and Cyclophosphamide
ANA: Antibodies to nuclear antigens
PMO: Post-menopausal osteoporosis
ATN: Acute tubular necrosis
eGFR: estimated glomerular filtration rate
MDRD: Modification of diet in renal disease
